# Recent Advances in Proteomics and Cancer Biomarker Discovery

**DOI:** 10.4137/cmo.s539

**Published:** 2008-02-09

**Authors:** Gary Guishan Xiao, Robert R. Recker, Hong-Wen Deng

**Affiliations:** 1 Osteoporosis Research Center, Departments of Medicine and Biomedical Sciences, Creighton University, 601 N 30^th^ ST, Suite 6730, Omaha, NE 68131; 2 Departments of Orthopedic Surgery and Basic Medical Sciences, University of Missouri—Kansas City, 2411 Holmes Street. Room M3-C03, City, Missouri, 64108-2792

**Keywords:** biomarker, genomics, proteomics, cancer

## Abstract

Early diagnosis and prevention is a key factor in reducing the mortality and morbidity of cancer. However, currently available screening tools lack enough sensitivity for early diagnosis. It is important to develop noninvasive techniques and methods that can screen and identify asymptomatic patients who have cancer. Biomarkers of cancer status can also serve as powerful tools in monitoring the course of cancer and in determining the efficacy and safety of novel therapies. Thus, discovery of novel specific biomarkers are needed that may provide informative clues for early diagnosis and treatment of cancer. Recently, remarkable progress has been made in the development of new proteomics technology. The progress that has been made in this field is helpful in identifying biomarkers that can be used for early diagnosis of cancer and improving the understanding of the molecular etiological mechanism of cancer. This article describes the current state of the art in this field.

## Introduction

Cancer is the second leading cause of death in the United States (1). It is estimated that more than 11 million people are diagnosed with cancer every year (1). This number is estimated to rise to 16 million every year by 2020 (1). Early diagnosis and prevention are key factors needed to reduce the mortality and morbidity of all types of cancer. Unfortunately, currently available cancer screening tools (e.g. mammography and invasive needle or surgical evaluation for breast cancer; or chest X-ray for lung cancer, etc.) are not sensitive enough for early detection of the disease. It is imperative to develop non-invasive techniques that distinguish between patients with and without cancer, as well as between stages of cancer.

Recently, genomic and proteomic technologies have evolved rapidly in cancer research. Genomic technologies allow us to monitor thousands of gene expression profiles simultaneously and evaluate interactions of candidate genes to obtain a global view of cancerous tissue in a single unbiased experiment. Despite its ubiquity and remarkable usefulness, microarray technology has technical limitations because transcriptional regulation is often difficult to reconcile with protein abundance, and the transcriptome poorly correlates with the proteome in a cell (2,3). Proteomics studies allow us to understand proteins and their modifications which may not be reflected by analysis of gene expression. The proteome contains all of the gene products that represent the functional output of a cell rather than nucleic acids that are derived from an individual’s full genetic code. Proteomics has now gained more attention because by directly analyzing protein expression at the post-translational level, it permits the qualitative and quantitative assessment of a broad-spectrum of proteins that can be related to specific cellular responses (4–6). Proteome analysis provides useful clues to biological processes happening at their level of occurrence, allowing comparison of physiological and pathological states of a cell line or a tissue. Further, proteomics, as a “new genomics”, can be used not only to study expression profiling of the whole cell, but also can apply to study of cellular compartments and organelles and their time-resolved dynamics (7).

## Proteomics as a Powerful Biomarker Screening Tool

Proteomics is the large-scale study of proteins, particularly their structure and functions, including detection, identification, measurement of their concentration, characterization of modification, characterization of protein-protein interaction and regulation. This term was coined to make an analogy with genomics, but it is much more complicated than genomics. Most importantly, while the genome is a rather constant entity, the proteome is a rather dynamic entity and differs from cell to cell. The proteome in cells is constantly changing through its biochemical interactions with the genome and the environment. With completion of a rough draft of the human genome, many researchers are now focusing on how genes and proteins interact to form other proteins. It is estimated that the human proteome consist of 500,000 proteins derived from about 35,000 genes in the human genome. The large increase in protein diversity may be due to *alternative splicing* (8,9) and *post-translational modification* (10,11) of proteins. This discrepancy implies that protein diversity cannot be fully characterized by gene expression analysis alone, making proteomics a promising tool for characterizing cells and tissues of interest and for biomarker discovery. In addition, some proteins may be expressed during very short periods of time in the life of an individual, while others may be continually expressed but with half lives too short to be isolated and detected (12). Therefore, although much effort has been devoted to bio-marker discovery in clinical research, few effective biomarkers are available for early diagnosis of cancer (Table 1). In this communication, we will summarize advances in proteomics that might be used for efficient and effective cancer biomarker discovery. We will also attempt to shed light on future directions of proteomics research for cancer biomarker discovery.

## Qualitative Proteomics

Proteomics research includes the characterization of protein mixtures in order to understand complex biological systems and determine relationships among proteins, their functions, and protein-protein interactions. Generally, proteomics can be characterized as qualitative proteomics and quantitative proteomics. Qualitative proteomics experiments aim to study changes in protein expression (13). Mass spectrometry (MS)-based quantitative proteomics has become an increasingly popular approach to study changes in protein abundances and diversity in biological samples. Qualitative proteomics aims to monitor changes in protein mixture composition under different physiologically relevant conditions (14). Similar to genomics study, a classic qualitative proteomics study compares the relative levels of thousands of protein species in different biological samples by standard protein profiling technologies, such as protein microarrays, 2-DE, 2-DLC. Taking advantage of the genome sequence database, query algorithms and newly-developed mass spectrometry instruments, qualitative proteomics has been enhanced in characterizing the molecular mechanisms of diseases (15–17). Quantitative proteomics provides quantitative information for all proteins in a sample instead of only providing lists of identified proteins (18–19). It aims to discover differences between samples (e.g. healthy and diseased patients). The methods of protein identification are identical to those used in qualitative proteomics, but include quantification as an additional dimension.

### Two dimensional gel electrophoresis

Two-dimensional gel electrophoresis (2DE) is one of the most common proteomics technologies with relative low resolution and simple technique. Proteins are separated first based on charge across a defined PH gradient in one direction and then separated by mass in another vertical direction. It allows us to monitor thousands of proteins simultaneously in a semiquantitative manner and to detect the protein components of each spot by identifying discriminating spots from the gels robotically and analyzing their sequence by tandem mass spectrometric methods (4,5,20,21). 2D gel analysis is the most well established standard for protein profiling of complex protein mixtures. The most notable limitation is that 2-DE requires relatively large amounts of sample material and can only identify the most abundant proteins.^14^

### Two dimensional liquid chromatography

Although gel-based proteome profiling has been widely used for protein separation, it suffers from several significant shortcomings such as lack of throughput potential and reproducibility as well as difficulties in resolving proteins that are highly basic, or of high molecular weight, or in low abundance (22–25). To circumvent problems associated with 2-DE, several liquid-phase separation methods (26), such as size-exclusion chromatography (27), affinity chromatography (28) and ion-exchange chromatography (29) have been developed.

Combinations of the different liquid-phase separation methods can be formed for multiple dimensional liquid chromatography that are suitable for separation of the whole cellular proteome as well as the plasma proteome. Recently, a new multidimensional liquid chromatographic separation system (M-D LC) has been developed by combining chromatofocusing (CF) (30–32) and nonporous reverse phase column chromatography (NPRPC) (32–34). This new method can provide greater throughput potential for reproducible separation of complex mixtures in mammalian cells such as those in mouse macrophage cell lines (32–34). This method has some advantages such as high reproducibility between batches, but quantitative measurement of protein abundance in a sample remains a challenge.

## Quantitative Proteomics

While monitoring qualitative changes is valuable, We also need to develop quantitative tools that can provide deep insight into disease mechanisms in order to unveil key molecules that may play major roles in disease processing. Enabled by the advent of quantitative proteomics technologies, rapid advancements in global detection and quantitation of proteins have provided an enormous set of both opportunities and challenges to discover molecular mechanisms of cancer and other diseases. Great interest has been directed toward characterization of cell function (15), disease mechanism (16) and biomarker discovery (35). Recently, quantitative proteomics has been achieved by development of new strategies that use metabolic or post-extraction stable-isotope labeling alone, or in combination with affinity tags (12,36–38). In this section, we will summarize the development of quantitative proteomics methods.

### Radioactive labeling

Radioactive labeling is the most sensitive and reliable method to detect cellular protein dynamics. Briefly, protein is labeled with (35) S (39–40) or (32) P isotopes (41,42) and separated on 2-D gel. Labeled protein in the gel is then exposed to a storage phosphor screen, which is subsequently scanned with a laser. The protein detection limit is less than 1 pg. However, there is a trend toward replacing the radioactive material by using other labeling methods to avoid problems with radiation safety in the laboratory.

### Fluorescence labeling

To get similar sensitivity to radioactive labeling, fluorescence labeling provides a non-isotopic approach to study dynamic profiling of the proteome in cells or tissue. This labeling assay includes two-dimensional differential gel electrophoresis (2-DDGE).

#### Two-dimensional difference gel electrophoresis (2-DDGE)

2-DDGE is a protein differentiation technology that can execute a type of differential comparison of a given protein state in reference to a control. 2-DDGE differs from classic 2-DE in that the CyDye technology allows multiplexing a proteome display in one gel. This technology is not only a detection technique, but also offers a method for accurate quantitative proteomics. Using CyDye technology, different protein samples can be pre-labeled with dyes of different excitation and emission wavelengths, then mixed and run together in a single gel (14). Differentially expressed proteins can be subsequently identified by mass spectrometric methods. Although this technique shows some advantage in reducing gel-to-gel variation while compared to 2-DE, it also shows certain limitation for separation of proteins with high molecular weight, various hydrophobicity and extreme pI vaules.

### Stable-isotope labeling

Stable-isotope labeling can be classified as two types of methods, namely, chemical labeling and metabolic labeling in the living cell. The Chemical labeling method includes isotope-coded affinity tag (ICAT) technology (43–46) and isobaric tags for relative and absolute quantification (iTRAQ) (47–50). Metabolic labeling in living cells includes stable isotope labeling with amino acids in cell culture (SILAC) (48–50).

#### Isotope-coded affinity tags (ICAT)

ICAT, first developed by Aebersold and his colleges (36), is an innovative method of protein profiling that utilizes stable isotope labeling of protein samples from two different sources, which are chemically identical in all aspects other than isotope compositions. ICAT analysis profiles the relative amounts of peptides containing cysteine that are derived from tryptic digests of protein extracts. Proteins extracted from the two samples are labeled with either light or heavy ICAT reagents, and react via cysteinyl thiols on the proteins. Peptides are recovered by avidin affinity chromatography and are then analyzed by LC-MS-MS. This produces a full scan spectrum which displays the abundance of light and heavy peptide ions and their relative proteins.

The significance of ICAT technology is that it can be used to identify 300–400 proteins per sample without using the 2-D gel (43). Also, enrichment of low-abundance proteins can be performed before the analysis through cell lysate fractionation (44). ICAT technology has been widely used for protein identification and quantification in mammalian, liver and breast tumor cells (43). Disadvantages of ICAT analyses are; they are only applicable to proteins containing cysteine; they identify far fewer proteins than 2-DE; and they contain a large label, which makes database searching more difficult, especially for short peptides (44).

### Isobaric tags for relative and absolute quantification (iTRAQ)

iTRAQ have been newly developed by Ross et al (48) and first introduced by Applied Biosystems (Applied Biosystems, Framingham, USA). This method is used for multiplexed quantitative proteomic analysis (48) and applied to different applications of proteome profile analysis (45,49,50). The principle for iTRAQ is to use a set of isobaric reagents which are amine specific to identify and quantify simultaneously up to four different samples. The amine specificity of these reagents makes most peptides in a sample amenable to this labeling strategy with no loss of information from samples involving post-translational modifications, such as phosphorylation. In addition, the multiplexing capacity of these reagents allows for information replication within certain LC-MS/MS experimental regimes, providing additional statistical validation within any given experiment. However, this is a chemical labeling method, which might generate side products during labeling and cause some loss of analytic sensitivity. Therefore, it may be only suitable for mass spectrometry validation of biomarker candidates. It may not be suitable for proteome dynamic profiling studies.

#### Stable isotope labeling with amino acids in cell culture (SILAC)

**Figure 1:**
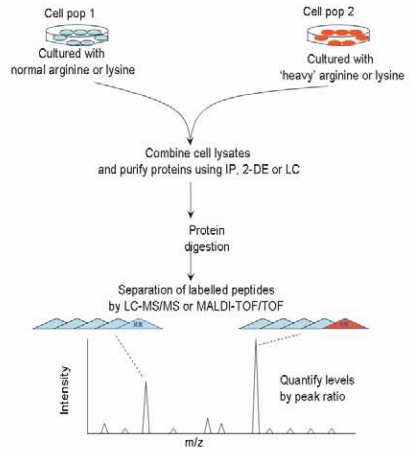
A schematic representation of the SILAC (‘stable-isotope labelling in cell culture’) method. A stably labelled amino acid in a cell-culture medium (in this case, ‘heavy’ arginine or lysine) is incorporated fully into the proteome of one cell population (“Cell pop 2”). Relative quantification experiments can easily be carried out using cells that were grown in normal media as the control (Cell pop 1). Cell lysates from two conditions can be combined and purified through many steps. The proteins are then digested and if the two forms of the peptides co-elute, a peptide ratio can be obtained for each mass spectrum, which allows the protein levels in the two populations to be quantified relative to each other.

SILAC has become a popular labeling strategy for peptide quantitation in proteomics experiments. It is a simple approach that incorporates a label into proteins for mass spectrometry (MS)-based quantitative proteomics *in vitro*. It was first developed by Mann et al (51) based on metabolic incorporation of a given ‘light’ or ‘heavy’ form of the amino acid into the proteins in living cultured cells. The method relies on the incorporation of amino acids with substituted stable isotopic nuclei (e.g. deuterium, ^13^C, ^15^N, ^18^O). Thus in an experiment, two cell populations are grown in culture media that are identical except that one of them contains a ‘light’ and the other a ‘heavy’ form of a particular amino acid (e.g. ^12^C and ^13^C labeled L-lysine, respectively) (Fig. 1). When the labeled analog of an amino acid is supplied to cells in culture instead of the natural amino acid, it is incorporated into all newly synthesized proteins. After a number of cell divisions (2 or more), each instance of this particular amino acid will be replaced by its isotope labeled analog. Since there is little chemical difference between the labeled amino acid and the natural amino acid isotopes, the cells behave exactly like the control cell population grown in the presence of normal amino acids. It is efficient and reproducible as the incorporation of the isotope label is 100%. This is a promising pioneer technique that can be used for characterization of phenotype-associated cellular signaling transduction. It is now being extensively applied for bio-marker discovery (35), cell signaling dynamics (52), identification of posttranslational modification sites (53,54), protein-protein interaction (55–57) and subcellular proteomics (58).

Gronborg et al. have used this strategy to study the differential secreted proteome in the case of pancreatic cancer (35). A human pancreatic ductal epithelial cell line was grown in normal media and the pancreatic cancer cell line was grown in media supplemented with heavy isotopic forms of arginine and lysine (^13^C_6_). The media were harvested, and proteins were resolved on a SDS PAGE. LC-MS/MS was further carried out following trypsin digestion. They successfully identified five confirmed proteins (CD9, perlecan, SDF4, apoE, and fibronectin receptor) as potential biomarkers that may be used for diagnosis of pancreatic cancer (35). A similar approach was employed by Yocum et al. to investigate possible protein signatures in different MLL leukemias in order to identify disease biomarkers and protein targets for pharmacological intervention using MV4 -11 and RS4:11 cells in culture (59). The majority of biomarkers and drug targets are membrane associated proteins. Recently, Liang et al. used SILAC to perform differential membrane proteomics in breast cancer cells to identify proteins that are differentially expressed on the surface of a breast cancer cell when compared to its normal counterpart (60). They have quantified 1600 gene products that group into 997 protein families with approximately 830 membrane or membrane-associated proteins. This study demonstrated that SILAC, a powerful technique, can be potentially useful for the discovery of membrane-bound antigens in phenotype-associated studies (60).

## Perspectives

Qualitative proteomics has provided very valuable information for understanding biological problems in the past decade. Proteomics technologies are the most important and useful approaches to observe and identify biomarkers with significant clinical meaning in cancer research. Protein biomarkers identified, for example, will help to improve the early diagnosis of cancer, provide a tool to monitor response to treatment and enhance the quality of patient administration (61). Protein biomarkers identified can also serve as therapeutic targets and provide mechanistic approach for effective drug design (61). Proteomics, however, still has many challenges in discovering tumor biomarkers. The challenge raised by quantitative proteomics is how best to identify large numbers of proteins from complex biological samples (Table 2). The multistep method appeared as a potentially powerful technique for large quantitative and qualitative proteomics research. Complex biological samples can be effectively divided into the relevant identification of increasing numbers of proteins. Currently, the major challenge is how to identify diagnostic patterns specific to cancer states from the huge dynamic range of biomarker concentration and biological variability among patient samples. For example, variation in sample collection, handling or storage and profiling techniques may influence the protein profile obtained from a given sample. So it is critical to solve and resolve these problems in biological variation, pre-analytical variation and analytical variability. It is increasingly recognized that routine proteomic analysis should be applied in the clinical setting to enhance reproducibility and validation of tumor biomarkers.

The popular iTRAQ method^48^ is a novel method that can offer quantitative measure of the cell proteome. In summary, this method can: 1) improve overall protein and proteome coverage while retaining important post translational modification information, 2) simultaneously compare multiple samples, e.g. normal versus diseased versus drug treatment samples, or apply to time course studies, all in the same experiment, 3) quantify and validate specific proteins of interest, such as biomarkers, or to screen drug targets and 4) increase statistical relevance needed for quantitative experiments by expanded multiplexing, up to four, to include duplicates or triplicates in the design. This is a chemical labeling method that can be quantitatively used for biomarker validation. Although iTRAQ can be done on any proteome including body fluids and biopsy material, it might also introduce side products (false positive results) that limit the sensitivity of the analysis since chemical strategies involve a derivatization step that might not be complete.

The SILAC method seems to be promising for study of biomarker discovery since it is based on metabolic labeling strategy in living cells. It offers a few advantages: 1) the expected mass difference is known before peptide identification, thus simplifying the quantitation, 2) mammalian cells are easily labeled by providing SILAC amino acids other than eliminating any unlabeled nitrogen source (e.g. if ^15^N is used) from the cultured medium, 3) a high degree of labeling since only one or two amino acids in a peptide can be substituted, 4) technically, quantitation is simplified and straightforward (64). Although the traditional isotope labeling method can not provide protein synthesis information, this approach allows for the determination of changes in protein expression levels of all cellular proteins by determining the mass spectral peaks corresponding to the unlabeled (from manipulated cells) to labeled (from control cells) protein as illustrated in equation (1).

(1) $$\text{Protein expression}=\frac{\left[\text{unlabeled peak}\right]}{\left[\text{labeled peak}\right]}.$$

A ratio of one means that protein is neither under- nor over-expressed. A ratio of <1 means under-expression (concentration is less than that of the control) and >1, over-expression (concentration is greater than that of the control). Because the doses used in this cell culture system (e.g. deuterium water used is >4%) are much higher than that usually used in the clinic (e.g. deuterium dose is <2%), application of this technology for clinical study has certain limitations. The existing algorithms of SILAC are incapable to handle such complex data generated from mass spectrometry in clinical patient study (65). To overcome the disadvantages mentioned-above, it is more important to improve analytic algorithms of MS data analysis. Interestingly, a recent patient study using deuterium water as a tracer suggests a promising algorithm for simplifying analysis of sophisticated MS data (65). This method, “modified SILAC (mSILAC)”, can measure protein synthesis rate quantitatively and protein turnover based on mass isotopmer distribution (MIDA) (65). The method can be applied to a large number of proteins (either known or unknown) in cells or tissues. This could improve the SILAC method that is used for the study of cell biology, including biomarker discovery.

In the future, a major concern will be about how to integrate effectively the proteomic with genomic and metabolomic, data and their functional interpretations in clinical results. In data analysis of proteomics in cancer research, with the rapid increase in cancer research dealing with datasets, exhaustive searching can not guarantee finding the best subset from the large numbers of variables. That means, statistically, we can exhaustively search all the potential combinations of the proteomic, genomic and metabolomic data, but one cannot guarantee all the combinations are true positive since there are multiple testing problems here if we want to test the “best” subset from large numbers of variables. Second, different variables may have interactions among them, thereby we need to develop robust multivariate method in order to investigate the correlations between different dataset and different variable within one dataset. Certainly, univariate approaches cannot handle the correlations between variables, resulting in losing important discriminatory information. Multivariate analysis and other approaches must be applied in the data analysis used in proteomics.

## Figures and Tables

**Table 1:** Summary of the tumor biomarkers identified by using proteomics.

Cancer type	Biomarker	Reference	Primary clinical use	Status	Sensitivity	Specificity
Bladder cancer	NMP22	(12)	Disease monitoring	Validated	Low	High
Breast cancer	CA15-3	(13)	Disease monitoring	Validated	Moderate	Poor
	CA27-29	(14)	Disease monitoring	Validated	-	-
	CEA	(15)	Disease monitoring	Validated	-	Low
	Her2/Neu	(16)	Disease monitoring	Validated	-	Moderate
Colorectal cancer	CEA	(17)	Disease monitoring	Validated	Moderate	Low
Esophageal	Periplakin	(18)	Disease monitoring	Validated	-	-
Gastrointestinal stromal tumor	CA19-9	(19)	Disease monitoring		-	Poor
Hepatocellular carcinoma	α-fetoprotein	(20)	Staging	Validated	-	Moderate
Leukemia	HnRNPs	(21)	Disease monitoring	Putative	-	-
Lung cancer	CEA	(22)	Disease monitoring	Validated		Low
	Epidermal GFR	(23)	Selection of therapy	Validated		Low
	Cyfra21-1	(24)	Disease monitoring	Validated	High	Very high
Lymphoma	Histone H4	(25)	Disease monitoring	Putative	-	-
Nasopharyngeal carcinoma	Serum amyloid A	(26)	Diagnosis	Putative	-	-
Ovarian cancer	Human chrionic gonadotropin-β	(27)	Staging	Validated	-	Low
	Apolipoprotein A1	(28)	Diagnosis	Putative	-	-
	Heptaglobin α-subunit	(29)	Diagnosis	Putative	-	-
	CA-125	(30)	Diagnosis	Putative	-	
	Transthyretin fragment	(31)	Diagnosis	Putative	-	-
	Osteopotin	(32)	Diagnosis	Putative	-	-
Pancreatic cancer	CA19-9	(33)	Disease monitoring	Validated	High	Poor
	α1-antitrypsin and α1-antichymotrypsin	(34)	Diagnosis	Putative	-	-
	Apolipoprotein A1	(35)	Diagnosis	Putative	-	-
	Heptaglobin α-subunit	(36)	Diagnosis	Putative	-	-
Prostate cancer	PSA	(37)	Selection of therapy	Validated	High	High
	Vitamin D-binding protein	(38)	Diagnosis	Putative	-	-
	Osteopotin	(39)	Diagnosis	Putative	-	-
Renal cancer	Serum amyloid alpha	(40)	Disease monitoring	Putative	-	-
Liver	AFP	(41)	Diagnosis	Validated	Moderate	-

**Table 2:** Summary of proteomics approaches for tumor biomarker discovery.

Approach type	Approach	Advantages	Disadvantages	Reference
Qualititative Analysis	Protein microarray	> good for unknown protein functional assay > high throughput	> limited information > relative expensive	(14)
	2-DE	> simultaneously monitor thousands of proteins > compatible with various stain methods > high throughput	> require relatively large amounts of starting material > only identify the most abundant proteins > not good reproducibility	(14)
	2-D LC	> greater throughput potential > good reproducibility > easy configure to MS analysis	> difficulty data analysis > nonquantitative > relative expensive	(32–34)
	MS-based proteomics	> highly sensitive > relative simple protocol > posttranslational modification analysis	> nonquantitative > too many redundant sequence	(62)
Quantitative Analysis	Radioactive labeling	> highly sensitive > very good quantitative > posttranslational modification analysis	> safety	(39–42)
	Fluorescence labeling	> highly sensitive > reduced 2-DE variation > compatible with MS analysis	> expensive > marginal reproducibility > only good for high abundance proteins	(14,63)
	ICAT	> highly sensitive > good quantitative	> limited application > difficulty data analysis	(44,36)
	iTRAQ	> good proteome coverage > simultaneously comparison of multiple samples > good statistic relevance > good quantitative and good for biomarker validation	> possible false positive > reduced sensitivity because of chemical labeling	(48)
	SILAC	> known expected mass difference prior to identification, simple quantition > highly labeling yield, easily labeling in mammalian cells > protocol simple and straightforward > highly sensitive > potential application *in vivo* study	> difficulty data analysis for low or partially labeled species	(35, 51–61, 64)
	mSILAC	> known expected mass difference prior to identification, simple quantition > highly labeling yield, easily labeling in mammalian cells > protocol simple and straightforward > highly sensitive > application for *in vivo and* cell culture studies	> to be validated	(65)
